# Effect of 100% Oxygen-Modified Atmosphere Packaging on Maintaining the Quality of Fresh-Cut Broccoli during Refrigerated Storage

**DOI:** 10.3390/foods12071524

**Published:** 2023-04-04

**Authors:** Yukexin Dai, Xiaoyan Zhao, Jinhua Zuo, Yanyan Zheng

**Affiliations:** 1 Institute of Agri-Food Processing and Nutrition, Beijing Academy of Agriculture and Forestry Sciences, Beijing Key Laboratory of Agricultural Products of Fruits and Vegetables Preservation and Processing, Key Laboratory of Vegetable Postharvest Processing, Ministry of Agriculture and Rural Affairs, Beijing 100097, China; 2 College of Food Science, Shenyang Agricultural University, Shenyang 110866, China

**Keywords:** high-oxygen MAP packaging, shelf-life, nutritional compounds, antioxidant capacity

## Abstract

The effect of 100% oxygen (O_2_)-modified atmosphere packaging (MAP) on the quality improvement of fresh-cut broccoli stored at 4 °C for 15 days was investigated in this study. The results indicated that, compared to the control group conditions, 100% O_2_ MAP treatment effectively maintained broccoli sensory evaluation scores, green color, and texture; reduced respiration and chlorophyll degradation; and reduced total bacterial count (TBC), malondialdehyde (MDA) levels, electrolyte leakage (EL), hydrogen peroxide (H_2_O_2_), and superoxide (O_2_^−^) contents. Furthermore, 100% O_2_ MAP led to a smaller loss of nutrients and increased antioxidant capacity. In conclusion, the use of 100% O_2_ MAP is an effective approach for maintaining high-quality fresh-cut broccoli during refrigerated storage at 4 °C.

## 1. Introduction

The demand for fresh-cut vegetables, especially fresh-cut broccoli, has increased due to the convenience, freshness, and nutritional value of fresh-cut vegetables [1]. Fresh-cut broccoli is highly nutritious, serving as an excellent source of vitamins, phenolic compounds, and glucosinolates, which have antioxidant, anticancer, and metabolic balance properties [2]. However, fresh-cut broccoli is prone to severe quality deterioration, such as that caused by chlorophyll degradation [3]; production of off-odors [4]; and loss of nutrients and antioxidants [5], which limit its market circulation and sales. Therefore, effective and safe technologies, such as voltage electrostatic fields [6], UV-C [7], ultrasonication [8], and selenium-chitosan [9], need to be developed in the fresh-cut broccoli industry. The use of modified atmosphere packaging (MAP) is becoming increasingly popular as a preservation technique in the fresh-cut vegetable industry due to its ability to extend the shelf-life of fresh-cut produce. By modifying the gas ratio within the packaging, MAP can help prevent the deterioration of fresh-cut vegetables and maintain their quality [10]. The technique is widely used in the preservation of various fresh-cut vegetables, including Chinese yam [11], cucumber [12], iceberg lettuce [13], potato [14], and lotus root [15]. Recently, high-oxygen MAP has emerged as a novel approach in maintaining the quality of fresh-cut vegetables. A study by Li et al. (2021) showed that using 80% O_2_ MAP can effectively minimize the loss of firmness, color, and nutrients in fresh-cut carrots [16]. Another study by Fan et al. (2016) found that 60% O_2_ MAP can reduce the accumulation of reactive oxygen species (ROS) and decreased flavor decay in fresh-cut vegetables [17]. Additionally, a report by Kader et al. (2000) suggested that high-level O_2_ MAP has the potential to alleviate oxidative damage in vegetables, resist microbial invasion, and preserve the fresh flavor and sensory quality of the produce [18]. These findings highlight the potential benefits of high-oxygen MAP in the fresh-cut vegetable industry and encourage further exploration of its use in preserving the quality of fresh-cut produce. However, no study has reported an investigation of the effect of high-oxygen MAP (100% O_2_) on fresh-cut broccoli.

The aim of this study was to investigate the effects of 100% O_2_ MAP on the visual appearance, microbial count, respiratory rate, nutritional quality, cell membrane integrity, reactive oxygen scavenging (ROS) system, and antioxidant activity of broccoli. This study is significant, as it aims to elucidate the impact of 100% O_2_ MAP on various aspects of fresh-cut broccoli during storage, which will provide valuable insights into the preservation of fresh-cut broccoli and help extend its shelf-life.

## 2. Materials and Methods

### 2.1. Materials

Broccoli (*Brassica oleracea* L.) was procured from an agricultural market in Beijing and immediately transported under refrigeration at 4 °C to the laboratory. Only broccoli heads that were free from yellowing, damage, and decay were selected for the study.

### 2.2. Sample Preparation

The broccoli heads were cut into uniform florets with a height of 4 cm and a weight of 10–18 g. They were then washed three times with clean water and centrifuged for 2 min at 4500 rpm. The broccoli florets were divided into two groups, each group with 32 polyethylene terephthalate (PET) boxes, containing 200 g of broccoli. The boxes were connected to an atmosphere packaging machine (YZ-600, Yizun Biotechnology Company, Shenzhen, China). The control group (CK) samples were directly sealed and packaged, while the 100% O_2_ group samples were filled with 100% O_2_ and then sealed before being stored at 4 ± 0.5 °C with 90–95% humidity for 15 days. Sampling was done every 3 days, and one box was used for quality determination, including appearance, firmness, color, sensory scores, total bacterial count, electrolyte leakage (EL) rate, headspace gas, and respiration rate. The remaining two boxes of broccoli florets were frozen using liquid nitrogen for subsequent analysis of the remaining indices. Unless specifically stated in the measurement method, the indicators were measured three times (*n* = 3).

### 2.3. Visual Appearance, Color, Sensory Quality

The color of five broccoli florets was measured using a colorimeter (CM-700d, Konica Minolta Co., Osaka, Japan), with a single experiment conducted. The color parameters, L* (lightness) and a* (reddish-greenish), were determined. The visual appearance of four representative broccoli florets was captured using a camera (EOS80D, Canon Digital Co., Ltd., Tokyo, Japan) in a single experiment.

The sensory quality of the broccoli florets was evaluated by ten trained panelists, who judged changes in color, flavor, structural state, decay rate, and flower bud opening degree. The visual quality was assessed using a 10-1 hedonic scale, as described by Alvarez et al. (2019), where 10 represents “extremely fresh and marketable”, 7 represents “inferior fresh” (minimal quality change), 5 represents “limit of marketability”, and 1 represents “extremely unacceptable” [19]. The experiment was conducted once.

### 2.4. Gas Composition

The concentrations of O_2_ and CO_2_ in each package were determined three times using a gas analyzer (Oxybaby 6.0 O_2_/CO_2_, MAPX 4.0, Rome, Italy). To ensure accurate measurements, the sample was inserted into the package before analysis.

### 2.5. Texture Analysis

The texture of the broccoli stem was analyzed using a texture analyzer (Stable Micro Systems, Surrey, UK). A cylindrical probe with a 5 mm diameter and 5 g trigger load was used, with a pretest speed of 1 mm/s, test speed of 1 mm/s, posttest speed of 10 mm/s, and a 7 mm distance between the probe and the sample [20]. The texture analysis was performed once on five broccoli florets, and each stem was measured at two points. The results were reported as force (N).

### 2.6. Respiration Rate

The respiration rate was measured at 4 °C by a respiration analyzer (GXH-3059, Junfang Scientific Instrument, Beijing, China) according to Lv et al. [21]. Fresh-cut broccoli (200 g) was placed in a 1.5 L sterile glass container. The measurement was performed three times, with intervals of 30 min, 10 min, and 10 min. The respiration rate (Q) was expressed as the release amount of CO_2_ kg^−1^ h^−1^.

$$Q=\frac{F\times 60\times C}{22.4}\times \frac{44}{W}\times 10^{-6}\times \frac{273}{273+T}$$

where the *F*, *C*, *W*, and *T* represent the flow rate of 500 mL min^−1^, CO_2_ concentration, sample weight, and temperature of 4 °C, respectively.

### 2.7. Electrolyte Leakage (EL) Rate and Malondialdehyde (MDA) Content

Briefly, 15 g of florets were immersed in 35 mL distilled water for 1 h (represented as EI_0_). The sample was then heated at 100 °C for 10 min and cooled to obtain EI_1_. The EL was calculated as EI_0_/EI_1_%.

The MDA content was assessed according to the methods of Xie et al. [22]. The mixture of 2 mL trichloroacetic acid solution (0.6% *w*/*v*) and 2 mL sample supernatant was boiled at 100 °C for 10 min. The absorbance was measured at 450 nm, 532 nm, and 600 nm.

$$\mathrm{MDA}\left(\mathrm{nmol}\;g^{-1}\;\mathrm{FW}\right)=\left[6.45\times \left(A_{532}-A_{600}\right)-0.56\times A_{450}\right]\times Vt/(Vs\times m)$$

where *A*_450_, *A*_532_, and *A*_600_ represent the absorbance at 450, 532, and 600 nm, respectively. *Vt* and *Vs* represent the total volume of the extract solution and volume of extract solution involved in the reaction, respectively, and represents potato sample quality [22].

### 2.8. Total Bacterial Count (TBC)

The TBC was determined according to Park et al. [8]. First, 25 g of fresh-cut broccoli sample were mixed with 225 mL of 0.1% sterilized peptone water (Biolink Microbiology Technology Co., Ltd., Guangdong, China) and homogenized in a sterile filter bag (Bi Teman Biological Science and Technology Ltd., Shanghai, China) for 3 min. A 1 mL aliquot of the supernatant was diluted in 9 mL of 0.1% peptone water with a 10-fold gradient. An amount of 100 μL of the diluted supernatant was carefully placed on agar medium (Bi Teman Biological Science and Technology Ltd., Shanghai, China) and incubated at 37 °C for 48 h.

### 2.9. Total Chlorophyll and Total Carotenoid Content

The total chlorophyll and carotenoid content was determined according to the methods of Xu et al. [23]. First, 0.5 g of frozen broccoli was homogenized with a mixture of acetone and ethanol (2:1) for approximately 20 min at 4 ℃. The total chlorophyll content absorbance was measured at 645 nm, and the carotenoid content was determined at 663 nm.

### 2.10. Total Glucosinolates and Sulforaphane

The total glucosinolate content was assessed according to the methods of Mawlong et al. [24]. A frozen broccoli sample was extracted with 80% methanol for 30 min. The reaction system consisted of 100 μL of supernatant, 250 μL of distilled water, and 3 mL of 2 mM sodium tetrachloropalladate. The total glucosinolate content was measured at 425 nm.

The sulforaphane content was determined according to the methods of Huang et al. [25]. The extraction was carried out using dichloromethane, anhydrous sodium sulfate, and a rotary evaporator (MLC3, Heidolph Instruments GmbH & CO. KG, Schwabach Municipality, Germany). The sulforaphane content was measured by high-performance liquid chromatography (HPLC) (1260, Agilent Technologies, California, USA) using a C18 reversed-phase column.

### 2.11. Vitamin C (VC) Content

The VC content was assessed according to the methods of Xu et al. [23]. To extract the VC, 1 g of broccoli was mixed with 5 mL of oxalic acid-EDTA in the dark for 10 min and then centrifuged. The reaction system was made up of 2 mL of the supernatant, 3 mL of oxalic acid-EDTA, 0.5 mL of metaphosphoric acid-acetic acid, 1 mL of 5% H_2_SO_4_, and 2 mL of 5% ammonium molybdate solution. The absorbance was measured at 750 nm.

### 2.12. Total Phenolic (TP) Content

The total phenolic (TP) content of broccoli was determined according to the methods of Blainski et al. [26]. Three grams of frozen broccoli were homogenized with 6 mL of precooled ethanol. The reaction system consisted of the sample supernatant (0.5 mL), 15 mL of distilled water, 1.25 mL of 0.5 mol/L Folin-Ciocalteu reagent, and 25 mL of 20% Na_2_CO_3_ solution. The mixture was incubated at 75 °C for 10 min and then measured at 760 nm. The standard curve was based on gallic acid, and the total phenolic content was expressed as gallic acid equivalents per gram of fresh weight (mg/g FW).

### 2.13. Superoxide (O_2_^−^) and Hydrogen Peroxide (H_2_O_2_) Content

The superoxide (O_2_^−^) content was determined according to the methods of Fan et al. [17]. The procedure involved incubating 2 mL of the supernatant with a mixture of hydroxylamine hydrochloride (10 mmol/L), sulfanilic acid (17 mmol/L), and α-naphthol (7 mmol/L) for 10 min. The absorption was recorded at 525 nm.

The hydrogen peroxide (H_2_O_2_) content was determined according to the methods of Lanubile et al. [27]. A 3 g frozen sample was homogenized with 6 mL precooled acetone. The extracted solution (1 mL) was mixed with a mixture of 0.1 mL 5% Ti(SO_4_)_2_, 0.2 mL concentrated NH_4_OH solution, and 3 mL of 18 mol/L H_2_SO_4_. The absorbance was read at 405 nm.

### 2.14. Measurement of Antioxidant Activity: DPPH, ABTS, SOD, and CAT

The measurement of antioxidant activity was performed as follows: 

DPPH: The assay was carried out based on the methods of Ancos et al. [28]. One hundred microliters of extract were mixed with 900 μL of 0.5 mM DPPH solution, and the absorbance was measured at 517 nm.

ABTS: The assay was performed according to the methods of Re et al. [29]. An amount of 25 μL of sample extract was added to 2 mL ABTS solution and reacted in the dark for 6 min. The absorbance was measured at 734 nm using Trolox solution for the standard curve.

SOD: The activity of SOD was measured using a spectrophotometry method [30]. The reaction mixture consisted of 45 μL of 100 μmol/L EDTA-Na2 solution, 100 μL of 750 μmol/L nitroblue tetrazolium solution, 3 μL of xanthine oxidase, 35 μL of 130 mmol/L methionine solution, and 18 μL of the crude enzyme extract. The absorbance was read at 560 nm.

CAT: The activity of CAT was determined based on the methods of Sun and Li [31]. One gram of frozen tissue was homogenized in 5 mL of 0.5% *w*/*v* polyvinyl polypyrrolidone solution to prepare the crude enzyme solution. The reaction mixture consisted of 1.9 mL PBS (pH 7.8, 0.1 M), 1 mL H_2_O_2_ (0.3% *w*/*v*), and 0.1 mL enzyme extract, and the change in absorbance was recorded at 240 nm.

### 2.15. Statistical Analyses

The experiments were conducted three times, except where otherwise specified. Statistical analysis was performed using IBM SPSS 27 and Origin 2011. ANOVA was used to establish a 95% confidence interval. Different letters present in a single figure indicates a statistically significant difference (*p* < 0.05).

## 3. Results and Discussion

### 3.1. Effect of 100% O_2_ MAP on Gas Composition and Respiration Rate of Fresh-Cut Broccoli

**Figure 1:**
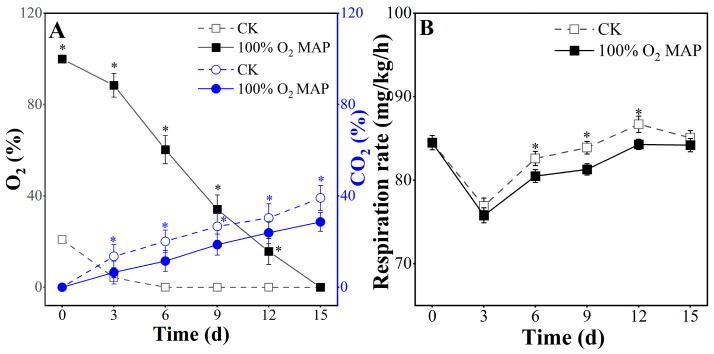
Effect of 100% O_2_ MAP on the gas concentrations of O_2_ and CO_2_ (**A**) and respiration (**B**) of fresh-cut broccoli. * in the figure indicates significant differences among treatments at the same storage time (*p* < 0.05). The error bar represents the standard deviation, and the data in the figure are displayed in the form of average.

After cutting, potatoes are still alive, and breathing inevitably consumes O_2_ and releases CO_2_ in the packaging. The gas composition in the package can reflect the metabolic rate of fruits and vegetables. In this study, the concentration of O_2_ and CO_2_ inside the package over the course of 15 days at 4 °C was monitored and is shown in Figure 1A. Both 100% MAP and the control group (CK) showed a declining trend in O_2_ and an increasing trend in CO_2_, which was consistent with the findings of Barbosa et al. [32]. During the storage period from day 0 to 12, the 100% O_2_ MAP group maintained a higher concentration of O_2_ and a lower concentration of CO_2_ compared to the control group. However, the control group had a detrimental gas environment with low O_2_ and high CO_2_, leading to unpleasant flavors. The respiration rate is shown in Figure 1B. During storage, respiration rate initially decreased after day 3, increased after day 6, and decreased after day 12; however, the respiration rate in 100% O_2_ MAP was slightly lower than that of control group.

Anaerobic respiration, caused by a low O_2_ and high CO_2_ environment, limits the quality of vegetables during storage [33]. Additionally, an increase in respiration due to a higher metabolic rate can result in decay and senescence of fruits and vegetables [34]. Bal (2013) found that vegetables with lower respiration rates have a longer shelf-life compared to those with higher respiration rates [35]. The findings of these studies strongly support the conclusion that 100% O_2_ MAP creates a favorable gas packaging environment and regulates the respiratory metabolism of broccoli.

### 3.2. Effect of 100% O_2_ MAP on the Appearance, Color, Chlorophylls, and Carotenoids of Fresh-Cut Broccoli

**Figure 2:**
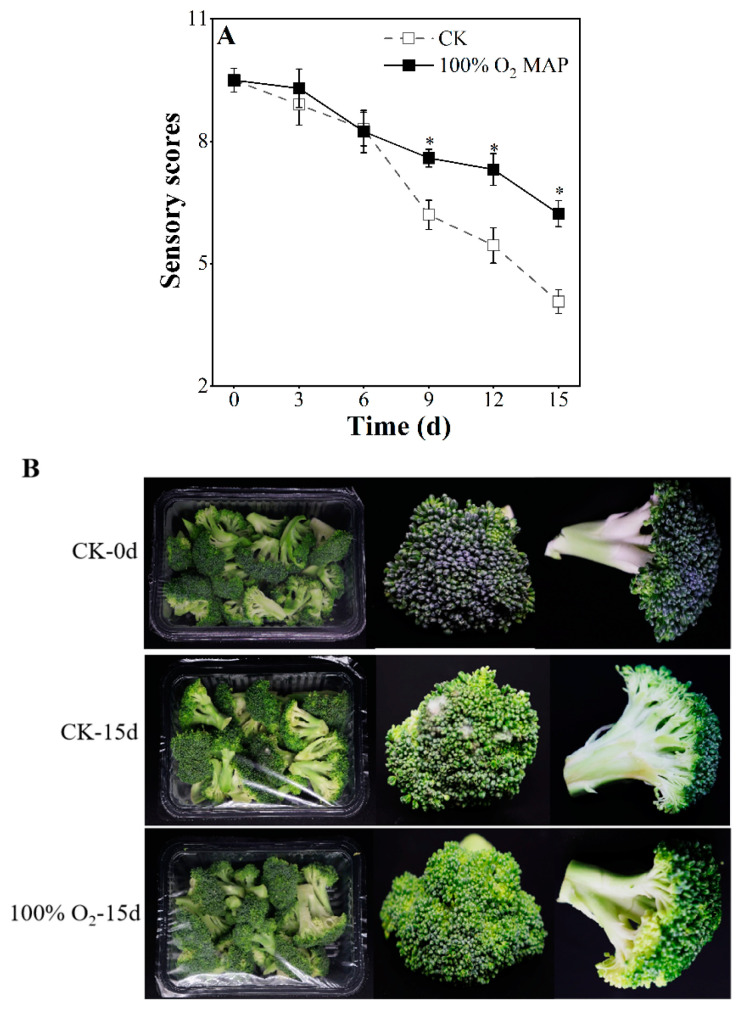
Effect of 100% O_2_ MAP on representative sensory scores (**A**), visual photograph (**B**), of fresh-cut broccoli. * in the figure indicates significant differences among treatments at the same storage time (*p* < 0.05).

The visual quality of fresh-cut broccoli is a crucial factor in its commercial appeal. The effects of a high O_2_ atmosphere on broccoli’s appearance and sensory scores were investigated and are shown in Figure 2. Both the control and 100% O_2_ MAP-treated broccoli showed a decrease in sensory scores as storage time progressed (Figure 2A). The control group showed signs of commercial decline by the 12th day (sensory score 5.47) and was no longer acceptable by the 15th day (sensory score 4.07). In contrast, the 100% O_2_ MAP group had a higher sensory score and was still acceptable at the 15th day of storage (sensory score 6.23), indicating that 100% O_2_ MAP helped maintain the commercial viability of broccoli. On the 15th day of cold storage, most of the fresh-cut broccoli in the control group appeared to have decayed and had a peculiar smell, which contributed to its loss of commercial value. However, the fresh-cut broccoli in the 100% O_2_ MAP group did not decay, and the smell of broccoli slightly faded, but it was still acceptable (Figure 2B).

**Figure 3:**
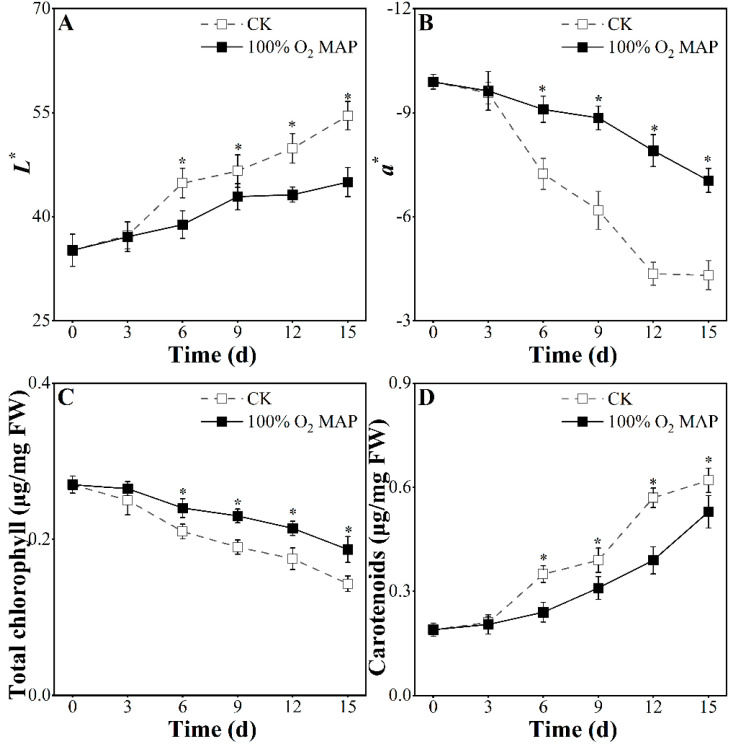
Effect of 100% O_2_ MAP on L* (**A**), a* (**B**), chlorophyll (**C**), and carotenoids (**D**) of fresh-cut broccoli. * in the figure indicates significant differences among treatments at the same storage time (*p* < 0.05).

Color change is a key sensory index for evaluating the freshness of broccoli [36]. The effect of color, chlorophyll, and total carotenoids is shown in Figure 3. As shown in Figure 3A,B, the L* and a* values increased during storage but were lower in the broccoli treated with 100% O_2_ MAP after the sixth day (*p* < 0.05). In this study, 100% O_2_ MAP effectively delayed the increase in L* and a* values, maintaining the green coloration of the broccoli, which is consistent with Wang et al. (2022) [37]. The biosynthesis of pigments, mainly chlorophyll and carotenoids, drives the greening and yellowing of broccoli, respectively [38]. A gradual decrease in chlorophyll and increase in carotenoids were observed in the control group, as shown in Figure 3C,D, indicating a loss of the original green color. However, broccoli treated with 100% O_2_ MAP maintained the total chlorophyll content and had an improved synthesis of carotenoids. Overall, the 100% O_2_ MAP group maintained lower L* and a* values by inhibiting chlorophyll degradation and carotenoid accumulation.

### 3.3. Effect of 100% O_2_ MAP on the Microbiological Quality of Fresh-Cut Broccoli

**Figure 4:**
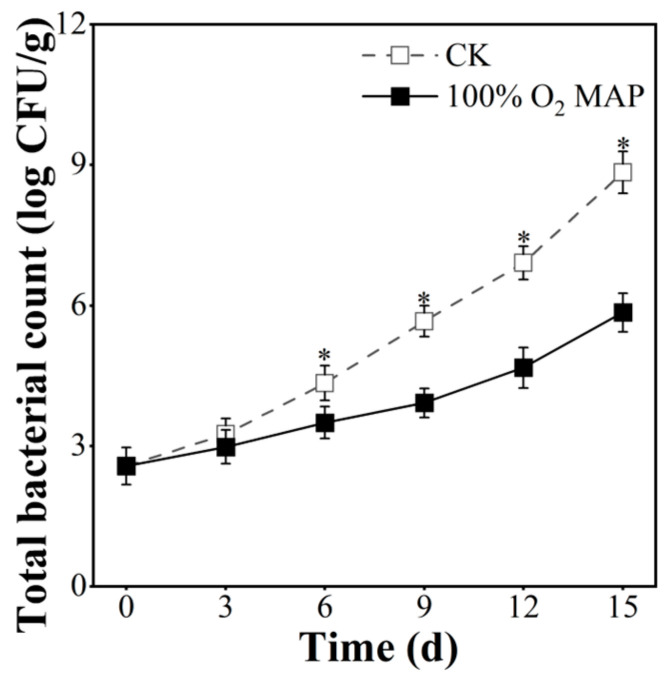
Effect of 100% O_2_ MAP on total bacterial count of fresh-cut broccoli. * in the figure indicates significant differences among treatments at the same storage time (*p* < 0.05).

The safety of fresh-cut produce is closely linked to the levels of microorganisms present, and thus, monitoring microbial count is critical [39]. In this study, total bacterial count (TBC) was used to evaluate the quantity of microorganisms (Figure 4), and the results showed that the TBC of fresh-cut broccoli consistently increased during the 12 days of cold storage. However, the TBC in the 100% O_2_ MAP group was significantly lower than in the control group from the sixth to fifteenth day (*p* < 0.05). Furthermore, in the 100% O_2_ MAP group, the TBC was kept below 5 log CFU/g until the twelfth day, while in the control group, the TBC exceeded 5.66 log CFU/g on the ninth day. These results suggest that the 100% O_2_ MAP treatment effectively inhibits microbial growth and reduces TBC levels.

### 3.4. Effect of 100% O_2_ MAP on Nutrients in Fresh-Cut Broccoli

**Figure 5:**
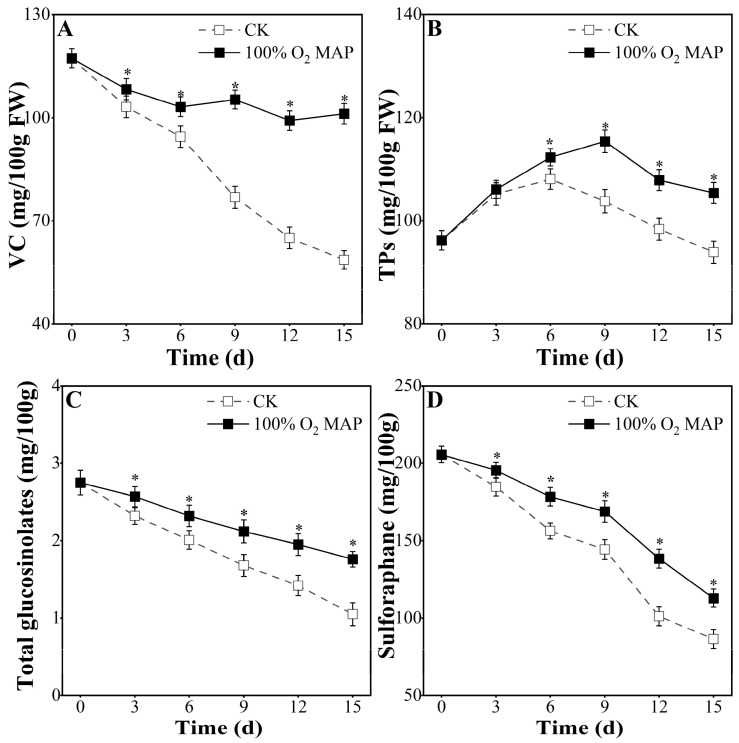
Effect of 100% O_2_ MAP on VC, TPs, total glucosinolates, and sulforaphane of fresh-cut broccoli; (**A**)—VC (mg/100 g FW); (**B**)—TPs (mg/100 g FW); (**C**)—Total glucosinolates (mg/100 g); (**D**)—Sulforaphane (mg/100 g) of fresh broccoli. * in the figure indicates significant differences among treatments at the same storage time (*p* < 0.05).

VC is an essential nutrient for humans that deteriorates over time. As shown in Figure 5A, the VC content decreased, but at storage day 15, the decrease was much faster in the control group compared to the 100% O_2_ MAP group (49.96% vs. 13.73%). This indicates that 100% O_2_ MAP effectively preserves the VC content in fresh-cut broccoli.

The TP content increased and then decreased (Figure 5B). During storage from the sixth to fifteenth day, the TP content in 100% O_2_ MAP was significantly higher than that in the control group. A study by Woods et al. (2007) showed that higher oxygen levels can enhance total phenol biosynthesis in blueberries [40], which is consistent with the 10.56% increase in TP content observed in 100% O_2_ MAP broccoli on the 15th day in the present study.

Retaining glucosinolates, a bioactive component in broccoli, is crucial for preserving its quality [41]. As shown in Figure 5C, total glucosinolate content declined during the 15 days of storage, which is consistent with Pe’rez et al. [42]. On the 15th day of storage, the total glucosinolate content in the 100% O_2_ MAP group was 0.64 times the initial value, while it was 0.38 times the initial value of the control group. The 100% O_2_ MAP group showed significantly higher total glucosinolate content than did the control group, indicating that 100% O_2_ MAP is effective in maintaining glucosinolate content in broccoli at 4 °C.

Sulforaphane (4-methylsulfinylbutyl isothiocyanate), a natural bioactive substance, is a compound with antioxidant and anticancer properties. Sulforaphane content decreased with storage time (Figure 5D), but the decrease was more pronounced in the control group. The sulforaphane content of the 100% O_2_ MAP group was 112.90 mg/100 g on the 15th day, which was 1.3 times higher than that of the control group (86.40 mg/100 g). There was a significant difference between the two groups from the third to fifteenth day of storage (*p* < 0.05). This suggests that 100% O_2_ MAP packaging helps preserve the sulforaphane content in broccoli.

### 3.5. Effect of 100% O_2_ MAP on Membrane Structure of Fresh-Cut Broccoli

**Figure 6:**
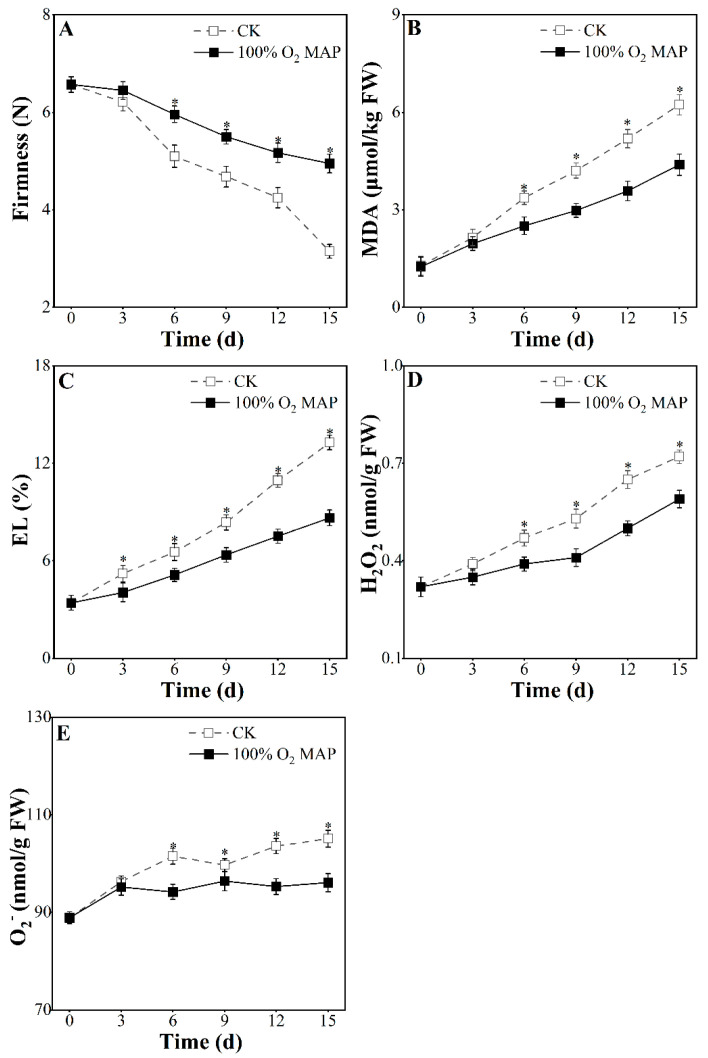
Effect of 100% O_2_ MAP on firmness (**A**), MDA (**B**), EL (**C**), H_2_O_2_ (**D**), and O_2_^−^ (**E**) of fresh-cut broccoli. * in the figure indicates significant differences among treatments at the same storage time (*p* < 0.05).

The parameters related to membrane integrity such as MDA, EL, H_2_O_2_, and O_2_^−^ are reported in Figure 6. As shown in Figure 6A, on day 15, the untreated group showed a higher loss of firmness (3.42 N) compared to the 100% O_2_ MAP group (1.62 N). This was likely due to less cellular tissue damage in the 100% O_2_ MAP treatment group, which is consistent with the findings of Huang et al. [43]. MDA is the final product of membrane lipid peroxidation, and EL reflects cell membrane permeability. As shown in Figure 6B,C, both the untreated and 100% O_2_ MAP groups showed a gradual increase in MDA and EL over time, but the increase was much slower in the 100% O_2_ MAP group, indicating its ability to reduce lipid peroxidation. When exposed to mechanical injury, the accumulation of oxygen species (ROS) increases, finally resulting in lipid peroxidation [44]. H_2_O_2_ and O_2_^−^ are involved in ROS metabolism, triggered by imbalances in ROS. The content of H_2_O_2_ and O_2_^−^ is shown in Figure 6D,E, with a serial upward trend observed in H_2_O_2_ and a fluctuant upward trend in O_2_^−^. The 100% O_2_ MAP group maintained significantly lower H_2_O_2_ levels from day six to fifteen (*p* < 0.05) and a significantly lower O_2_^−^ content on days six, twelve, and fifteen (*p* < 0.05), indicating its ability to minimize ROS metabolism.

Research has found that firmness loss is accompanied by membrane damage [45]. In this study, broccoli treated with 100% O_2_ MAP showed minimum values for MDA, EL, H_2_O_2_, and O_2_^−^, indicating reduced lipid peroxidation and minimized membrane damage, thereby preserving its firm texture.

### 3.6. Effect of 100% O_2_ MAP on Antioxidant Activity of Fresh-Cut Broccoli

**Figure 7:**
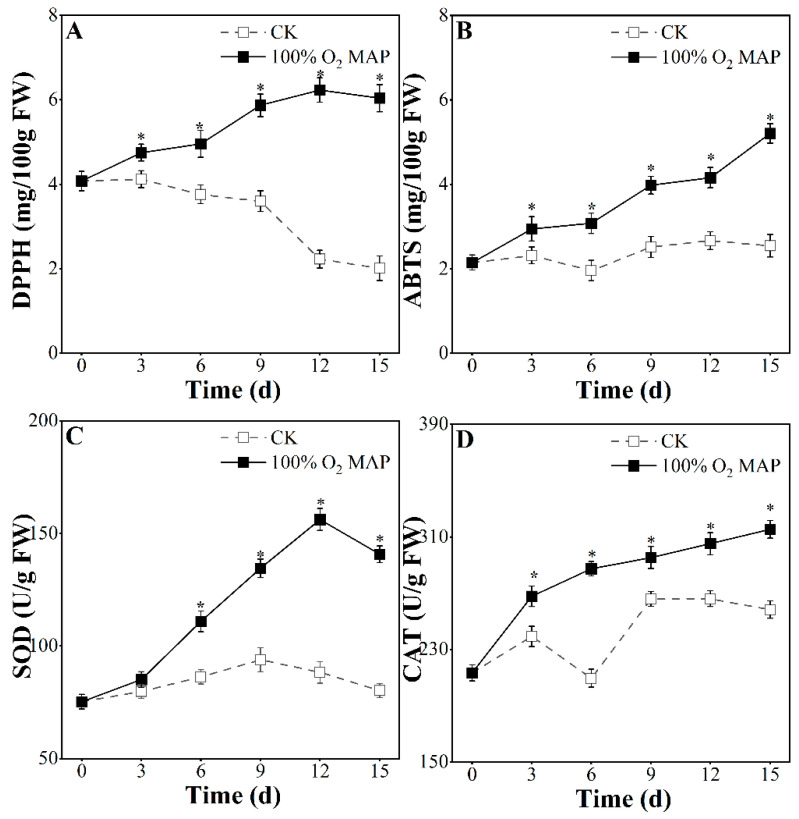
Effect of 100% O_2_ MAP on DPPH (**A**), ABTS (**B**), SOD (**C**), and CAT (**D**) in fresh-cut broccoli. * in the figure indicates significant differences among treatments at the same storage time (*p* < 0.05).

The parameters related to antioxidant capacity, such as DPPH and ABTS free radical scavenging activities, are reported in Figure 7A,B. During storage, the DPPH and ABTS scavenging capacity of the 100% O_2_ MAP-treated broccoli increased, while the control group showed a decrease or no change. The 100% O_2_ MAP treatment led to a 48.04% and 142.33% increase in DPPH and ABTS scavenging after eight days, respectively, while the control group showed a 50.74% and 3.8% decrease, respectively. These findings support the conclusion that 100% O_2_ MAP increases the antioxidant capacity by enhancing DPPH and ABTS free radical scavenging capacity.

The establishment of an antioxidant defense barrier in plants can delay ROS accumulation and improve antioxidation ability. The major nonenzymatic antioxidant enzymes include SOD and CAT, which are responsible for the decomposition of H_2_O_2_ [46]. To further elucidate the mechanism of antioxidant activity, we analyzed the antioxidant enzyme activity of SOD and CAT (Figure 7C,D). During storage, compared with the control group, the 100% O_2_ MAP treatment group had higher SOD and CAT activity. On the 15th day, the SOD and CAT activity of the 100% O_2_ MAP-treated broccoli was 1.75-fold and 1.22-fold higher, respectively, than on day zero. Other studies have reported a significant positive correlation between antioxidant enzymes and antioxidant capacity, which is due to the ROS scavenging ability [18]. In our study, 100% O_2_ MAP reduced ROS damage and enhanced the antioxidant defense system by increasing SOD and CAT enzyme activity.

## 4. Conclusions

In conclusion, this study reports on the effectiveness of 100% oxygen-modified atmosphere packaging (MAP) on extending the shelf-life of fresh-cut broccoli to up to 15 days at 4 °C. The results indicate that 100% O_2_ MAP effectively preserves the sensory quality and reduces lipid peroxidation and chlorophyll degradation while increasing bioactive compounds and antioxidant capacity and suppressing microbial growth. These findings have important implications for the food packaging industry and suggest that high-oxygen MAP is a valuable solution for the storage of fresh-cut broccoli. The food packaging industry is encouraged to further explore the potential of 100% O_2_ MAP for the preservation of fresh-cut broccoli.

## Data Availability

Data are contained within the article.
